# Supplementary material for: Prognosticators for Patients with Pancreatic Ductal Adenocarcinoma Who Received Neoadjuvant FOLFIRINOX or Gemcitabine/Nab-Paclitaxel Therapy and Pancreatectomy
Source: Cancers (Basel). 2023 May 4;15(9):2608. doi: 10.3390/cancers15092608 (PMC10177033; doi:10.3390/cancers15092608)
Supplement: Supplementary file 1 [file cancers-15-02608-s001.zip › cancers-2347026-supplementary.pdf]

Supplemental Table S1.

Clinicopathologic Characteristics of Patients Who Underwent Upfront Pancreatectomy.

Characteristics	N=166
**Gender**	
Female	70 (42.2%)
Male	96 (57.8%)
**Median age (range, year)**	64.2 (24.9-84.8)
**Type of Surgery**	
Pancreatoduodenectomy	141 (84.9%)
Distal pancreatectomy	23 (13.9%)
Total pancreatectomy	2 (1.2%)
**Lymphovascular invasion**	
Negative	40 (24.1%)
Positive	126 (75.9%)
**Perineural invasion**	
Negative	7 (4.2%)
Positive	159 (95.8%)
**Margin status**	
Negative	137 (82.5%)
Positive	29 (17.5%)
**pT stage**	
pT1	28 (16.9%)
pT2	113 (68.1%)
pT3	25 (15.1%)
**pN stage**	
pN0	42 (25.3%)
pN1	54 (32.5%)
pN2	70 (42.2%)
